# “Humanly Possible”: Geographies, Metrics and Methods to Address Immunization Inequalities

**DOI:** 10.3390/vaccines12091062

**Published:** 2024-09-18

**Authors:** Devaki Nambiar, Ahmad Reza Hosseinpoor, Nicole Bergen, M. Carolina Danovaro-Holliday, Ciara E. Sugerman, Hope L. Johnson

**Affiliations:** 1 Department of Data and Analytics, World Health Organization, 20 Avenue Appia, 1211 Geneva, Switzerland; nambiard@who.int (D.N.); bergenn@who.int (N.B.); 2 Department of Immunization, Vaccines, and Biologicals, World Health Organization, 20 Avenue Appia, 1211 Geneva, Switzerland; danovaroc@who.int; 3 Global Immunization Division, Global Health Center, Centers for Disease Control and Prevention, Atlanta, GA 30329, USA; bwf1@cdc.gov; 4 Measurement, Evaluation and Learning Department, Gavi, The Vaccine Alliance, 1218 Geneva, Switzerland; hjohnson@gavi.org

The year 2024 marks the 50th anniversary of the World Health Organization (WHO) Expanded Program on Immunization (EPI). WHO Director General Dr. Tedros Adhanom Ghebreyesus, acknowledging the incredible success of the EPI in showing what is “humanly possible”, called for the continued support and funding of initiatives to ensure that life-saving vaccines are available to all [1]. This occasion has renewed attention on immunization as a critical component of primary health care. Immunization remains a continuing priority as countries seek to safeguard the health of populations, as well as strengthen their health systems.

However, the gains made in the last 50 years are not evenly distributed across all populations. The COVID-19 pandemic has exposed and exacerbated inequalities of immunization coverage in critical childhood and adolescent vaccines, such as the diphtheria–tetanus–pertussis-containing vaccine (DTP) and the Human Papillomavirus vaccine (HPV), with past progress being lost in some cases [2]. Equity-sensitive and action-oriented framing, including the identification of Zero Dose (ZD) children, namely those who do not receive even one dose of essential life-saving immunization in childhood, is increasingly applied for both research and programming addressing immunization inequities [3,4]. ZD framing draws attention to the intersections of different dimensions of inequality and is one way in which inequities may be made visible and understood.

The 2023 Special Issue of Inequality in Immunization highlighted various forms of inequalities, drivers of these inequalities, and the impact of equity-focused interventions [2]. We presented emerging evidence on a range of data sources and dimensions of inequality, as well as processes by which inequalities are emerging. We were pleased to reprise these themes and also go beyond them in the 2024 Special Issue of Inequality in Immunization. This Special Issue comprises 14 contributions including 10 research articles, 2 reviews, a project report and a perspective piece.

This Special Issue covers a wide scope of contexts where inequalities are arising and being addressed. Johns et al. and Kalbarczyk et al. carried out ecological analyses of low- and middle-income country contexts. At the regional level, Castro-Aguirre et al. explored immunization inequalities in Latin America and the Caribbean, and Fahmy et al. conducted an analysis of the Eastern Mediterranean region. A scoping review by Lyons et al. captured research on childhood immunization inequalities globally.

This Special Issue also features two method-focused papers. Rachlin et al. drew attention to data triangulation and linking of datasets and databases to enable inequality analysis in Bangladesh, Rwanda and Nigeria, and Corrêa et al. demonstrated the use of targeted local surveys to track ZD children in ZD Learning Hubs established by Gavi in Bangladesh, Mali, Nigeria, and Uganda. Niyibitegeka et al. explored pneumococcal conjugate vaccine inequalities in terms of the distribution of economic benefits.

A number of country-focused papers are also included, seeking to deepen our understanding of within-country inequalities for key vaccines. Aheto et al. assessed routine immunization and ZD prevalence in Nigeria, Muhoza et al. reported on the second year of life and catch-up vaccination in Ghana, Woyessa et al. looked at measles vaccine uptake and barriers in Ethiopia, Mbunga et al. addressed ZD status in the Democratic Republic of the Congo (DRC), Meghani et al. studied adult COVID vaccination in India, and Olusanya et al. analysed paediatric COVID-19 and routine immunization in the United States.

As aforementioned, ZD framing is important to identify and reach populations that have not received any vaccinations owing to experiences of diverse and multiple forms of disadvantage. Four papers in this Special Issue—authored by Fahmy et al., Corrêa et al., Aheto et al. and Mbunga et al.—covered inequalities in the prevalence of ZD, adopting different definitions for ZD to align with the study and country context. For example, Mbunga et al., in the context of DRC, and Fahmy et al., in the context of the Eastern Mediterranean Vaccine Action Plan countries, adopted the Immunization Agenda 2030 definition for ZD, namely children who have not received their first dose of the Pentavalent vaccine (DTP-Hib-HepB) by the age of 12 months. Aheto et al., in their article featuring Nigeria, defined ZD as non-receipt of DTP, measles-containing vaccine, oral polio vaccine, and BCG among children aged 12 to 23 months. While the conclusions drawn largely cohere, i.e., low levels of maternal education and utilisation of services, remoteness and distance from facility were associated with greater ZD prevalence, an argument is made for slightly variable measurement approaches to enable operational relevance. For instance, Corrêa et al. explore measuring the timeliness of immunization and including older age cohorts. As underscored in the paper by Lyons et al., having a range of indicator variations, some providing immediate operational input and others allowing broader analytical understanding of trends and gaps, enables analyses to reflect different contextual considerations, enhancing their use and relevance.

Wealth and maternal education levels were some of most explored dimensions of inequality in immunization at global and local levels, as well as those with the largest magnitudes and statistical significance, as evidenced by Johns et al., Aheto et al. and Lyons et al. Geographic accessibility is another major driver of inequalities explored in multiple studies in this Special Issue (including Fahmy et al., Aheto et al. and Mbunga et al.), largely through spatial analysis methods. One paper by Kalbarczyk et al. covered gender as an influence on immunization, finding that lack of time as well as cost constraints faced by women are major barriers to routine immunization coverage in sub-Saharan Africa and South Asia. The gender dimension in inequality research is still understudied, particularly in relation to other dimensions of inequality.

Attitudes and hesitancy towards vaccination represent another emerging theme that is closely connected to inequalities. Mbunga et al. and Olusanya et al., in contexts as variable as DRC and the United States, respectively, showed that parental/care-giver attitudes towards vaccination appeared to be highly correlated to under-vaccination and, in the case of DRC, to non-receipt of vaccination. In India, Meghani et al. identified engagement with community leaders, targeted counselling and door-to-door visits as important ways of addressing vaccine hesitancy and increasing awareness. Overall, this driver of inequalities warrants greater attention and further study across diverse country contexts.

This Special Issue has also explored inequality dimensions of the pneumococcal vaccine, which has been recommended for over two decades. The vaccine exists in surplus globally, but it is not equitably available across countries. Using 2021 birth cohort estimates, Niyibitegeka and colleagues developed a model of the total social welfare associated with the vaccine, demonstrating a 45-fold return on investment for manufacturers on the one hand, with 2.5–6.0% (per sensitivity analysis) of the total global surplus going to low-income countries, as compared to over a third of the surplus going to high-income countries. While more evidence related to the pneumococcal vaccine using an equity lens is needed, existing analyses suggest the need for redoubled efforts to increase vaccine access, reflecting the intention and spirit of EPI.

To conclude, this Special Issue provides evidence that transformation is “humanly possible” in two ways. First, the contributions show that immunization inequalities reflect structural factors like maternal education, gender inequality, societal norms, and global financing. And yet, these structural factors are remediable, suggesting on the one hand that these are inequities, but on the other that there is a possibility, and indeed a responsibility, for “human” intervention to mitigate them. Second, methodological insights related to measurement of ZD, standardization of indicators, use of various data sources and analytical approaches suggest that we are capable of refining our analyses, finding the gaps, and advancing on the path towards equity. The first fifty years of EPI have shown that it is possible to make vaccines accessible to large numbers of people around the world; in the coming 50 years, it is our responsibility to make immunization equity not merely possible or probable, but assured.
